# School Studies

**Published:** 1857-07

**Authors:** 


### SCHOOL STUDIES.

HAD I the choice of only four things to be taught my children, they should be:

To sing well:

To read well:

To write well:

To sketch well.

Perfection in these will earn their possessor a maintenance in any country, and will enable him to amuse himself or entertain a company, whether it be under a rock in the desert, or upon a crag in the sea.

